# Electrospinning of PCL-Based Blends: Processing Optimization for Their Scalable Production

**DOI:** 10.3390/ma13173853

**Published:** 2020-09-01

**Authors:** Marina P. Arrieta, Adrián Leonés Gil, Maysa Yusef, José M. Kenny, Laura Peponi

**Affiliations:** 1 Instituto de Ciencia y Tecnología de Polímeros (ICTP-CSIC), C/Juan de la Cierva 3, 28006 Madrid, Spain; marrie06@ucm.es (M.P.A.); aleones@ictp.csic.es (A.L.G.); maysay.b@hotmail.es (M.Y.); jose.kenny@unipg.it (J.M.K.); 2 Facultad de Óptica y Optometría, Universidad Complutense de Madrid (UCM), Arcos de Jalón 118, 28037 Madrid, Spain; 3 Interdisciplinary Platform for Sustainable Plastics towards a Circular Economy-Spanish National Research Council (SusPlast-CSIC), 28006 Madrid, Spain; 4 Civil and Environmental Engineering Department, STM Group, University of Perugia, 05100 Terni, Italy

**Keywords:** electrospinning, poly(ε-caprolactone), blends, microcrystalline cellulose, poly(3-hydroxybutyrate), stretchability

## Abstract

In this work poly(ε-caprolactone) (PCL) based electrospun mats were prepared by blending PCL with microcrystalline cellulose (MCC) and poly(3-hydroxybutyrate) (PHB). The electrospinning processing parameters were firstly optimized with the aim to obtain scalable PCL-based electrospun mats to be used in the industrial sector. Neat PCL as well as PCL-MCC and PCL-PHB based mats in different proportions (99:1; 95:5; 90:10) were prepared. A complete morphological, thermal and mechanical characterization of the developed materials was carried out. Scanning electron microscopy (SEM) observations showed that the addition of PHB to the PCL matrix considerably reduced the formation of beads. Both the addition of MCC and PHB reduced the thermal stability of PCL, but obtained materials with enough thermal stability for the intended use. The electrospun PCL fibers show greatly reduced flexibility with respect to the PCL bulk material, however when PCL is blended with PHB their stretchability is increased, changing their elongation at break from 35% to 70% when 10 wt% of PHB is blended with PCL. However, the mechanical response of the different blends increases with respect to the neat electrospun PCL, offering the possibility to modulate their properties according to the required industrial applications.

## 1. Introduction

Electrospinning can be considered an easy and effective way to obtain polymeric fiber mats with dimensions from micro to nano range [1]. Its main drawback is the difficulty to transfer electrospun mat production from laboratory to industrial scale, mainly due to the low productivity efficiency of the electrospinning process (processing throughput of a few milliliters per hour per single emitter) [2], as well as the requirement to standardize different parameters involved during the electrospinning process. In particular, two classes of parameters have to be optimized when working with the electrospinning: the parameters belonging to the electrospinning process itself (i.e., electrical potential applied, flow rate of the solution and working distance) and those depending on the electrospun polymeric solution (i.e., concentration, additives, solvent type, viscosity, conductivity and surface tension) [3]. However, during recent years, the production of electrospun mats has been gradually transferred to industrial products. At present, there are many companies that produce several electrospinning setups that allow mass production of electrospun fibers, with output around 5000 m^2^ daily [4].

The electrospinning process allows the production of long and continuous ultrathin polymeric fiber mats from a polymeric solution subjected to high electric fields at room temperature. The electrospun polymeric mats present large surface areas and high porosity with small inter-fibrous pore size [5]. Polymeric electrospun mats are very versatile materials and they can be used for the production of monolayer and multilayer films and/or sandwich type composites [6,7,8,9]. Moreover, the electrospun mats can be easily modified by reinforcing the fibers with organic [10,11] and inorganic nanofillers [12,13,14] and metallic nanoparticles [15,16]. Therefore, the electrospun mats have gained interest in the industrial sector since they can be easily processed as multifunctional materials and can be applied in different industrial fields such as controlled release of drugs [13], antioxidants [10,17] and/or antimicrobial [17] agents; in addition they have piezoelectric properties [18] and shape memory performance [19,20].

Poly(ε-caprolactone) (PCL) is a semi-crystalline biocompatible and biodegradable linear aliphatic polyester that offers a unique combination of polyolefin-like mechanical properties and polyester-like hydrolysability [21]. PCL is also recognized as safe by the Food and Drug Administration (FDA), providing high interest in several industrial applications such as the medical sector (e.g., medical devices, wound dressings, resorbable membranes, etc.), cosmetics, and in a number of short term applications (i.e., filtration membranes, films for packaging and agricultural applications) [22,23,24,25]. Furthermore, PCL presents a melting temperature (*T*_m_) around 60 °C and a glass transition temperature (*T*_g_) well below room temperature (around −60 °C), thus at room temperature it behaves as a flexible plastic [21]. However, the low melting temperature restricts its use as a mono-component in most applications [23,24]. Thus, many strategies have been proposed to improve PCL’s performance in order to extend its industrial applications; proposed improvements include copolymerization [21,22,23,24], the addition of different modifiers (e.g., fillers, nanofillers, etc.) [16] and blending with other biopolymers [24,26]. In this context, to guarantee the biocompatible and/or biodegradable character of the final PCL-based material, the polymer or additives added should also be biocompatible and biodegradable.

In this regard, cellulose and its derivatives are considered optimal materials for modifying biopolymers since they are bio-based, biodegradable, biocompatible, stiff, lightweight, non-abrasive to the processing equipment, highly abundant in nature and low in cost [27,28]. In fact, cellulose is the most abundant renewable polymeric resource available on earth and native cellulose is one of the strongest and stiffest natural fibers available, with a theoretical modulus estimated at 167.5 GPa [28]. Microcrystalline cellulose (MCC), obtained from high quality wood pulp by acid hydrolysis to remove the amorphous regions, is commercially available and its dimensions are in the range of 10 to 50 µm with a high specific surface area compared to other conventional cellulose fibers [28].

The family of poly(hydroxyalcanoate)s (PHAs) has gained considerable industrial attention since they are considered the most promising biopolymers to replace polyolefins [2]. PHAs are isotactic semi-crystalline high molecular weight polyesters biologically synthesized by controlled bacterial fermentation and further accumulated by the bacterial cell [29]. Poly(3-hydroxybutyrate) (PHB) is the simplest and most common representative of PHAs; it is highly crystalline and relatively comparable in certain physical properties to isotactic polypropylene (iPP) and polystyrene (PS) [2,29]. Garcia-Garcia et al. blended PCL with PHB, covering the full range between individual polymers at 25 wt % increments, and observed that although PCL and PHB are not fully miscible, some interactions between them occur [30]. However, the main drawback of PHB for its industrial transformation into PHB-based products is its narrow processing window [29]. Thus, the use of PHB for the production of electrospun mats allows extending its industrial exploitation since it is processed at room temperature, avoiding thermal degradation [2,31]. Moreover, electrospun fibers obtained by blending PHB with PLA reduce and/or avoid fiber defect formation (i.e., beads) since the highly ordered stereochemical structure of PHB leads to a highly crystalline polymer that, when it is added in low amounts, is able to produce uniform, straight and bead-less electrospun fibers [10,31].

**Figure 1:**
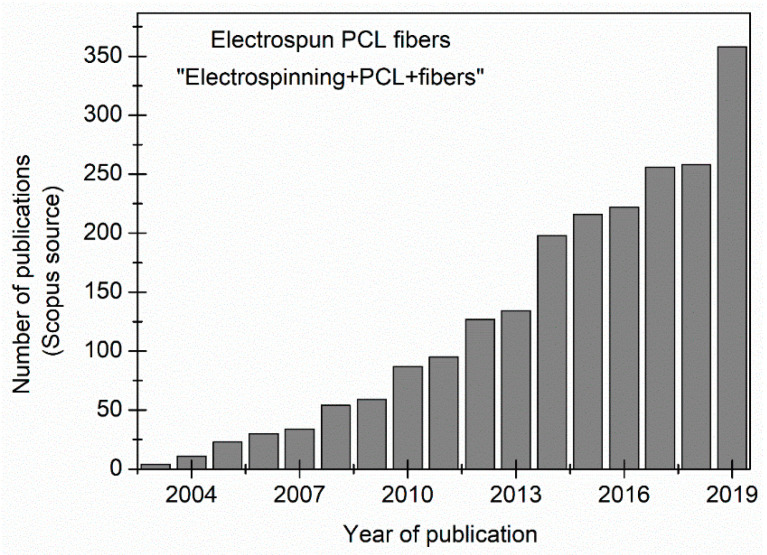
Number of publications on electrospun poly(ε-caprolactone) (PCL) fibers in the last 20 years (Scopus source).

In recent years the number of publications on PCL electrospun fibers has greatly increased, as reported in Figure 1. In fact, looking for the keywords “electrospinning” and “PCL” and “fibers” and considering only electrospun PCL-based mats it is worth noting that in 2019 more than 350 scientific papers were published on electrospun PCL fibers, underlying the strongly growing interest on these electrospun materials.

However, one of the main disadvantages when working with electrospun fibers is the poor mechanical properties of this material. In order to improve the mechanical performance of electrospun mats, several strategies have been reported, mainly focused on reinforcing biopolymeric matrices with both organic and inorganic nanofillers [16]. In 2018, Kai et al. [32] added lignin copolymers as a filler of nanofibers and obtained an increase in the tensile strength and elongation at break. Another more recent strategy was reported in 2020, in which the mechanical properties of electrospun PHB/PCL mats were modulated by studying different setup configurations (drum, blade and grid collector) in the electrospinning technique and incorporating post-processing thermal treatment. They improved the mechanical properties of the mats by heating near the melting temperature of PCL crystal, enabling the crosslinking of the fibers while preserving the fibrous structure [33].

Khalf et al. reported an interesting study on the use of triaxial electrospinning to obtain hollow fibers based on PCL [34]. Moreover, the miscibility of PCL with different biodegradable polymers was also studied in recent years [24,30].

Therefore, considering the need to gradually transfer the PCL-based electrospun fibers from the lab level to industrial products, systematic studies to standardize the processing conditions of PCL-based electrospun fiber blends are reported in this study, blending PCL with MCC or with PHB, both being bio-based polymers, named PCL-MCC and PCL-PHB, respectively. Considering that to prepare solvent-based electrospun polymeric fibers, polymers should be homogeneously dissolved in a proper solvent [31], the solvents were selected on the basis of a solubility parameter (δ) similar to that of the polymeric matrix. In particular, the PCL-based polymer solution concentration was set at 10 wt % in a solvent mixture of chloroform and dimethylformamide in 80:20 proportion on the basis of our previous works [5,16]. The PCL-MCC and PCL-PHB formulations were prepared in different proportions by adding 1, 5 and 10 wt % of MCC or PHB with respect to PCL. Firstly, the processing conditions were optimized with the aim to standardize the processing of PCL-based electrospun fiber mats for further industrial production. Then, the morphological aspect as well as the dimension of the PCL-based electrospun fibers were studied by scanning electron microscopy (SEM). Moreover, the solubility parameters of each component in the blend were compared to predict the miscibility of PCL with MCC and/or PHB. At the same time, the thermal behavior and mechanical properties of the electrospun fiber blend mats have been studied and compared with those of neat PCL. In particular, their thermal stability was evaluated by thermogravimetric analysis and their crystallinity behavior was studied by differential scanning calorimetry as well as by X-ray diffraction. Finally, their mechanical performance was evaluated by tensile test measurements in order to produce PCL-based electrospun blends providing the opportunity to tune their properties by simply varying their blend compositions, to be able to easily transfer the materials developed here to the industrial sector.

## 2. Materials and Methods

### 2.1. Materials

Poly(ε-caprolactone) (PCL, CAPA 6500, Mn = 50,000 g/mol, 0.5 wt % ε-caprolactone monomer) was kindly donated by Perstorp (Malmö, Sweden). Poly(3-hydroxybutyrate) (PHB, under the trade name P226, Mw = 426,000 Da) was supplied by Biomer (Kailling, Germany). Microcrystalline cellulose (MCC, dimensions of 10–15 μm) was supplied by Sigma-Aldrich (Madrid, Spain). Chloroform (CHCl_3_, 99.6% purity, boiling point 60 °C) and dimethylformamide (DMF, 99.5% purity, boiling point 153 °C) were supplied by Sigma Aldrich (Madrid, Spain).

### 2.2. Electrospun PCL-Based Mats Preparation

**Scheme 1:**
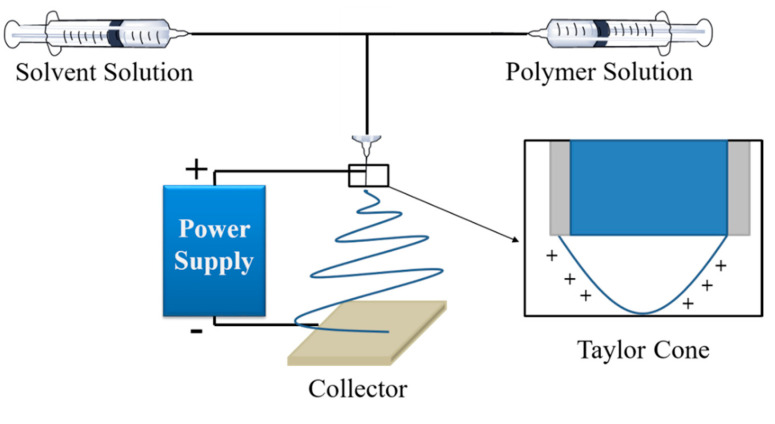
Schematic diagram of electrospinning in coaxial configuration.

Electrospun solutions based on neat PCL and PCL-based blends of PCL-PHB and PCL-MCC were prepared at 10 wt % in a solvent mixture of CHCl_3_:DMF (4:1) under a magnetic stirrer for 24 h at room temperature, on the basis of our previous work. Materials were pre-dried to remove absorbed water. PCL pellets were pre-dried under vacuum at room temperature for 24 h [24], PHB pellets were pre-dried at 40 °C for 4 h [31] and MCC powder was pre-dried at 80 °C in an oven overnight [35]. The electrospun PCL-based fiber mats were prepared by means of a coaxial electrospinning Y flow 2.2.D-XXX (Nanotechnology Solutions) with a vertical standard configuration. In Scheme 1 an example of the coaxial electrospinning used is reported. In our case, the diameter of the inner spinneret is 1.4 mm and of the external one is 1.7 mm.

The electrospun PCL-based mats were randomly collected in a grounded aluminum foil perpendicular collector; a working distance between the needle and the collector was set at 15 cm on the basis of previous works [5,16]. The polymeric PCL-based solutions were flowed through the inner needle and the same mixture of solvent used for the polymeric solutions (CHCl_3_:DMF in 4:1 proportion) was flowed through the outer needle, to avoid the evaporation of the polymeric solution on the external surface of the spinneret. In order to obtain the electrospun mat with a thickness of about 25–50 µm, the electrospinning was carried out during 3 h [31]. The polymer flow rate and the solvent flow rate varied between 0.5 and 5 mL/h; the applied positive and negative voltages varied from 1 to 10 kV, in order to optimize the processing window. Before starting the electrospinning process, the PCL-PHB as well as the PCL-MCC polymeric solutions were sonicated for 10 min in order to improve their dispersion and ensure homogeneity. The obtained electrospun PCL-based mats were vacuumed for 48 h in a vacuum chamber to eliminate any potential residual solvents and then were stored in a desiccator before characterization. The mat formulations and the proportion of each component as well as their designations are summarized in Table 1.

### 2.3. Electrospun PCL-Based Mats Characterization

The morphology of the electrospun PCL fibers were observed using a PHILIPS XL30 scanning electron microscope (SEM) (Phillips, Eindhoven, The Netherlands)). Samples were previously sputtered with a gold/palladium layer. Optical microscopy using a M568E Nikon Eclipse optical microscope (Nikon Corporation, Tokyo, Japan) at 100× magnification, equipped with a Nikon sight camera, was used to characterize the electrospun samples. The fiber diameters were statistically calculated from the SEM images with ImageJ software (version 1.51k, Java 1.6.0_24, Wayen Rasband, US National Institutes of Health, Bethesda, MD, USA), with 50 measurements for each sample. Thermogravimetric analysis (TGA) measurements were performed in a TA-TGA Q500 thermal analyzer (TA Instruments, New Castle, DE, USA) under dynamic mode. Electrospun PCL-based mats were heated from 30 to 700 °C at 10 °C/min under a nitrogen atmosphere. The onset degradation temperatures (*T*_5%_) were taken at 5% of mass loss and the maximum degradation temperature (*T*_max_) was obtained from the first derivative of the TGA curves (DTG).

Dynamic differential scanning calorimetry (DSC) experiments were performed in a Mettler Toledo DSC822e instrument (Mettler-Toledo, Schwarzenbach, Switzerland) under nitrogen atmosphere (50 mL/min). About 4 mg of each mat were sealed in aluminum pans. Each mat was firstly cooled from room temperature to −90 °C and then heated to 200 °C. The glass transition temperature (*T*_g_) was taken at the midpoint of the heat capacity changes. The melting temperature (*T*_m_) was determined and the degree of crystallinity (χ_c_) was calculated through Equation (1):

(1) $$\chi_{c}=100\%\times \left[\frac{∆H_{m}}{∆H_{m}^{c}}\right]\times \frac{1}{W_{\mathrm{PCL}}}$$

where Δ*H*_m_ is the melting enthalpy, $∆H_{m}^{c}$ is the melting heat associated with pure crystalline PCL (148 J/g) and 1/*W*_PCL_ is the proportion of PCL in the blend formulation [21].

The crystalline profile of PCL-based electrospun mats was examined with X-ray diffraction (XRD) equipment (BRUKER D8 Advance X-ray diffractometer, Karlsruhe Germany). Scanning was performed on square mat surfaces (10 mm × 10 mm). The diffraction patterns were obtained from a diffractometer using Cu Kα radiation at 40 kV with a scanning step of 0.02° between 5° and 50°, with a collection time of 10 s per step.

The mechanical properties of the PCL-based electrospun mats were evaluated by tensile test measurements conducted at room temperature with an Instron dynamometer (model 3366, INSTRON, Norwood, MA, USA) equipped with a 100 N load cell, at a crosshead speed of 10 mm/min and an initial length of 30 mm. Dog-bone style samples were used and at least five specimens were statistically analyzed by one-way analysis of variance (ANOVA) using OriginPro 8.1 software. To identify which groups were significantly different from other groups, mean comparisons were done employing a Tukey’s test with a 95% confidence level.

## 3. Results and Discussion

### 3.1. Optimization of the Electrospinning Processing-Window

Firstly, the neat PCL solution was processed into electrospun fibers, varying electrospinning processing conditions as is summarized in Table 2. Each run with the corresponding electrospinning processing conditions was used to produce randomly oriented electrospun PCL fibers during 30 s, while the formation of fibers was corroborated by SEM analysis. In the Supporting Information Figure S1, optical images of the beads formation in the electrospun fibers are reported. At the same time, the fiber diameter distributions corresponding to the runs in which the fibers are obtained are reported in the Supporting Information Figure S2.

**Figure 2:**
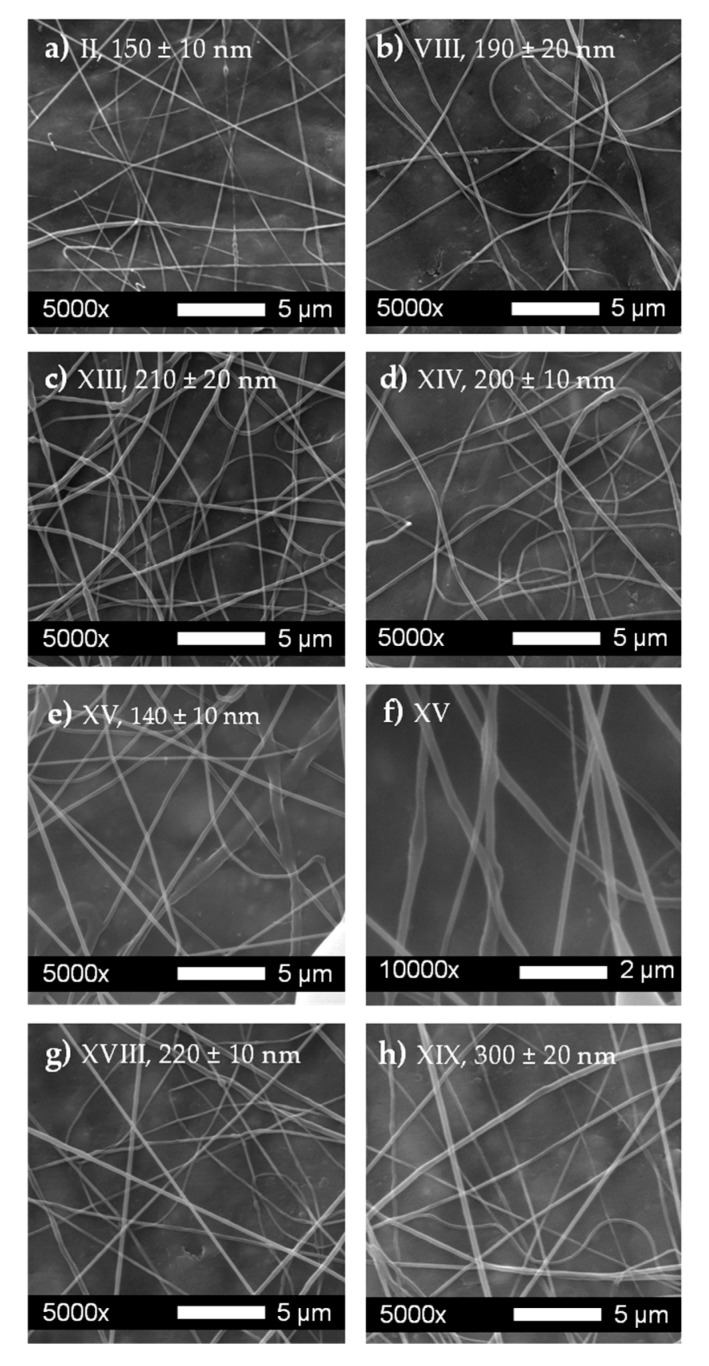
Scanning electron microscopy (SEM) images of electrospun PCL fibers and their average diameters corresponding to different runs of Table 2: (**a**) Run II, (**b**) Run VIII, (**c**) Run XIII, (**d**) Run XIV, (**e**) Run XV, (**g**) Run XVIII and (**h**) Run XIX. The image (**f**) corresponds to Run XV at a higher magnification of 10,000×.

Different positive and negative voltages (*V*, ranging from 1 to 10 kV) were assayed. It is known that to ensure the formation of the so-called Taylor cone that leads to the formation of electrospun fibers without defects, higher Coulomb forces are needed and thus higher voltages should be applied. The polymer flow rate (*Q*_p_) and the solvent flow rate (*Q*_s_) were varied from 0.5 to 5 mL/h. According to Table 2, there were seven possibilities of fiber formation, therefore in Figure 2 the SEM images of the electrospun fibers obtained with these conditions are reported.

### 3.2. Electrospun PCL-MCC and PCL-PHB Mats Characterization

To further produce the PCL-MCC electrospun blends, those conditions that allowed obtaining the three smallest fiber diameters for neat PCL were selected, that is Runs II, VIII and XV from Table 2. The selected conditions were those with polymer and solvent flow rates of 1.0 mL/h. In this sense, although Runs XIII and XIV also produced relatively thin fibers with average diameters in the range of 210 ± 20 nm, those conditions were discarded. Low polymer flow rates (such as in Run XIII, *Q*_p_ = 0.5 mL/h) require too much time to produce mats with thickness of about 25–50 µm. On the contrary, a low solvent flow rate (such as in Run XIV, *Q*_s_ = 0.5 mL/h) was not enough to favor the processing of electrospun PCL mats during 3 h, which was the selected time to produce mats with the required thickness of 25–50 µm. Thus, PCL99-MCC1’s proportion was selected to continue the optimization of electrospinning processing conditions. When PCL-MCC was processed with the conditions of PCL Run II (Table 2, Run II: *Q*_p_ = 1 mL/h, *Q*_s_ = 1 mL/h, *V*^+^ = 4 kV and *V*^−^ = 4 kV) and run VIII (Table 2, Run VIII: Q_p_ = 1 mL/h, Q_s_ = 1 mL/h, V^+^ = 10 kV and V^−^ = 4kV) no fibers were formed. Nevertheless, with the processing conditions of Run XV (Table 2, run XV: *Q*_p_ = 1 mL/h, *Q*_s_ = 1 mL/h, *V*^+^ = 10 kV and *V*^−^ = 10 kV) fibers were properly formed. Thus, these processing conditions were tested to process PCL99-PHB1, which successfully works. Hence, these processing conditions *Q*_p_ = 1 mL/h, *Q*_s_ = 1 mL/h, *V*^+^ = 10 kV and *V*^−^ = 10 kV were considered as optimum and used during 3 h to process all the formulations into electrospun mats.

**Figure 3:**
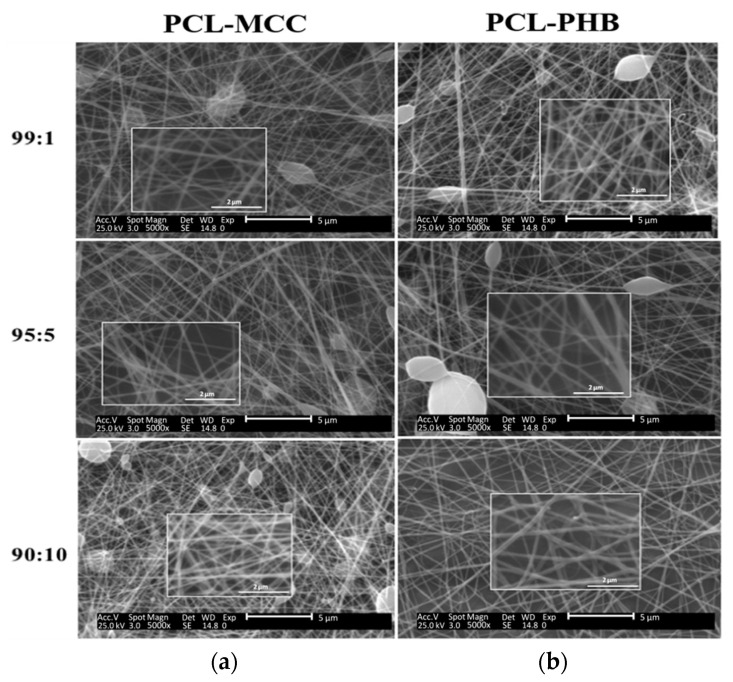
SEM images of (**a**) electrospun PCL-MCC mats and (**b**) PCL-PHB mats. Note: MCC = microcrystalline cellulose; PHB = poly(3-hydroxybutyrate).

The morphological aspects and the average diameter of the electrospun fibers of the PCL-MCC and PCL-PHB blends were studied by SEM (Figure 3). The corresponding fiber size distribution of the blend formulations is reported in the Supplementary Information in Figure S3. In Figure S4 of the Supporting Information, neat PHB fibers obtained by electrospinning and their fiber size distributions are also reported [31].

The fibers were generally straight in both PCL-MCC and PCL-PHB mats. The incorporation of MCC to PCL mainly maintains the PCL average fiber diameter and produces the formation of a few fiber defects (beads). The average fiber diameter slightly increases with an increasing amount of MCC. A small but the highest amount of beads was observed for the PCL90-MCC10 formulation, suggesting that 10 wt % of MCC is too high a concentration. In the PCL polymeric solution with the highest amount of MCC, cellulose fibrils can interact among themselves due to their tendency to establish hydrogen bonding interactions, leading to the aggregation of MCC, which obstructs the spinneret head producing discontinuous fiber deposition during the electrospinning process. The incorporation of 1 wt % of PHB to the PCL matrix led to the formation of thinner fibers than those with PCL99-MCC1, but also showed some bead defects. With an increase in the amount of PHB, the beads decreased and disappeared in the PCL90-PHB10 formulation. The higher amount of bead formations in the PCL-MCC mats can be also related to the higher differences in the solubility parameters of MCC (δ_MCC_ = 25.0 MPa^1/2^–30.2 MPa^1/2^) [36] with PCL (δ_PCL_ = 15.9 and 21.2 MPa^1/2^) [16,37] compared with PHB (δ_PHB_ = 18.5–20.1 MPa^1/2^) [38]. The ability of PHB to produce straight and bead-less electrospun fibers has already been observed in bio-polyesters blended with PHB such as poly(lactic acid) (PLA). The good miscibility between these two biopolyesters has also been ascribed to the similarity in their solubility parameters [31].

**Figure 4:**
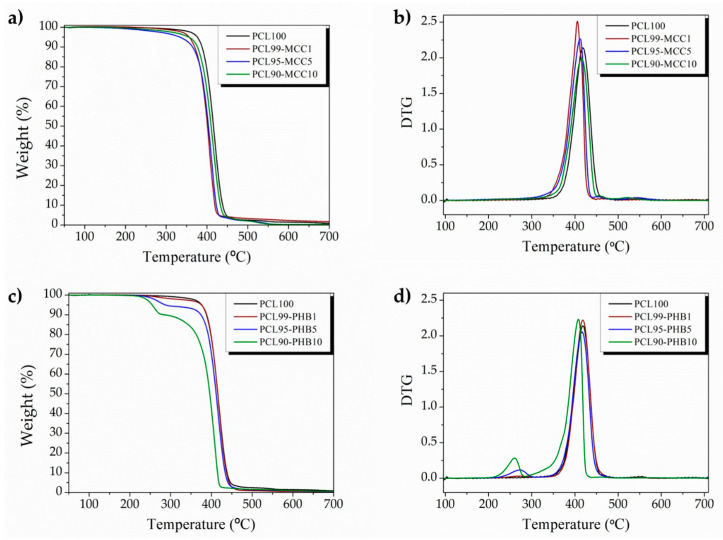
Thermogravimetric analysis (TGA) of electrospun PCL-MCC and PCL-PHB mats: (**a**) TGA curve of PCL-MCC, (**b**) DTG curve of PCL-MCC, (**c**) TGA curve of PCL-PHB and (**d**) DTG curve of PCL-PHB.

The effect of MCC and PHB addition on the thermal properties of the electrospun PCL-based mats was studied by thermogravimetric measurements. Figure 4 shows the TGA curves (Figure 4a,c) with the corresponding DTG curves of PCL-MCC (Figure 4b) and PCL-PHB (Figure 4d) electrospun mats, while the main thermal parameters obtained from these curves are summarized in Table 3.

The addition of both MCC and PHB decreased the onset degradation temperature (*T*_5%_), but showed considerably higher thermal stability than the processing conditions used here as well as for the intended uses such as medical devices, membranes for wound dressing, filtration membranes, films for agricultural or packaging applications, etc. In fact, the electrospun mats based on blends are thermally stable up to 280 °C. The neat PCL electrospun mat degrades in a single degradation process, showing the maximum degradation temperature (*T*_maxPCL_) at 419 °C. PCL-MCC blends also degrade in a single step degradation process. However, a shift of *T*_max_ to lower temperatures was gradually observed with increasing amounts of MCC in the blend. In fact, a decrease of about 10 °C was observed for PCL90-MCC10. Moreover, the degradation step starts firstly in PCL-MCC since MCC degradation occurs at a lower temperature (*T*_max_ of neat MCC = 323 °C) than the neat PCL matrix. The degradation of MCC is delayed in the PCL-MCC blends, suggesting that positive interaction is taking place between PCL and MCC. Two main interactions have been ascribed between PCL and MCC in the solid state [39]. Firstly, hydrogen bond interactions between the PCL carbonyl groups and hydroxyl groups from MCC, which are the responsible for delaying the MCC dehydration reaction which takes place at lower temperatures than depolymerization [35,39]. Then, as degradation proceeds, the effect of the above mentioned interactions decreases, but char and gases evolved from cellulose degradation may interact with solid PCL [39]. Different behavior is found for PCL-PHB mats. In fact, PCL-PHB electrospun blend formulations showed two thermal degradation processes, the first one related to the PHB (*T*_maxPHB_) degradation and the second one related to the PCL decomposition (*T*_maxPCL_), confirming that PCL is characterized by a remarkably higher thermal stability than PHB [30]. The *T*_maxPCL_ slightly decreased with an increasing amount of PHB. Nevertheless, some positive interactions between both polymers can be ascribed since the T_maxPHB_ was shifted from 258 °C in the neat PHB electrospun mat [10,30] to higher values (Table 3). This finding suggests that although the *T*_maxPHB_ changes in a very narrow range, PCL stabilized the PHB matrix in the PCL-PHB blends, slightly improving its thermal stability as was observed in the extruded PCL-PHB blends [30].

**Figure 5:**
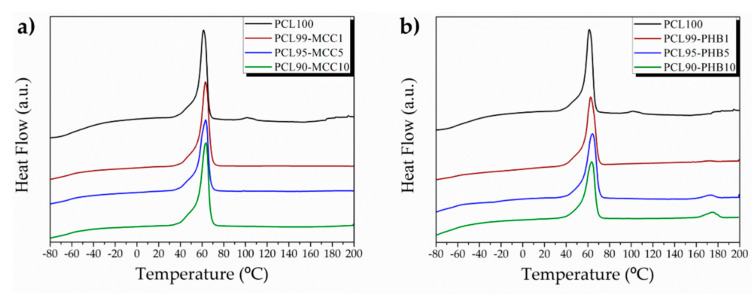
DSC curves of the first heating scan of electrospun (**a**) PCL-MCC and (**b**) PCL-PHB mats.

DSC analysis was conducted and the first heating scan curves are reported in Figure 5, while the DSC thermal parameters obtained from these curves are summarized in Table 3. *T*_g_ values of PCL practically did not change in PCL-MCC and PCL-PHB electrospun blends. Typical values of *T*_g_ for PHB are located in the 2–7 °C range [30], but no clear evidence of *T*_g_ is detected in PCL-PHB electrospun formulations (Figure 5b). The PCL melting temperature suffered minor increases in PCL-MCC and PCL-PHB compared with the neat PCL mat. While only one melting peak was observed in the PCL-MCC blends (Figure 5a), two individual peaks located at the typical temperature of each melting point were observed in the case of PCL-PHB blends (Figure 5b), suggesting the immiscibility between PCL and PHB in the blends [30]. Thus, in PCL-PHB blends one melting peak was observed at slightly higher temperatures than that of the neat PCL and the other melting peak, located at about 170 °C, was attributable to the PHB polymer [10,31], which slightly increased with an increasing amount of PHB in the PCL-PHB formulation. These slight variations of the two melting peaks suggest rather positive interactions between both polymers. However, in both electrospun blends the PCL seems to drive the crystallization process during the electrospinning process. In fact, the degree of crystallinity of the neat PCL was quite high (≅54%), indicating that the balance between the electrospun fiber formation and the solvent evaporation produced a quite crystalline material and the addition of MCC or PHB did not alter the high degree of crystallinity of the neat PCL [16]. This behavior has been already observed in our previous work in which electrospun PCL was embedded with several organic and inorganic nanoparticles, obtaining nanocomposites with a similar degree of crystallinity as that of the neat electrospun PCL [16].

**Figure 6:**
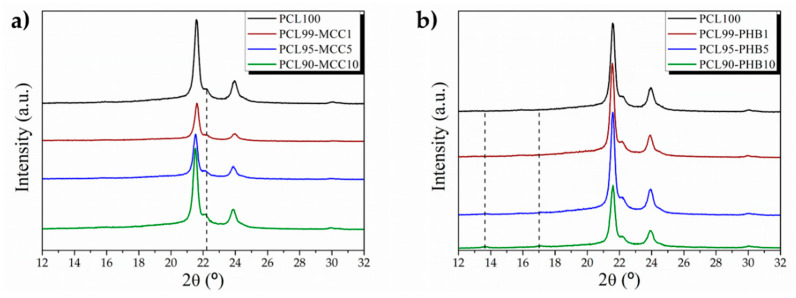
X-ray diffraction (XRD) curves of electrospun (**a**) PCL-MCC and (**b**) PCL-PHB mats.

The DSC findings were corroborated by XRD analysis (Figure 6), in which it can be seen that no significant changes were produced in the PCL diffraction pattern due to the incorporation of both MCC and PHB.

The diffraction patterns of PCL show the peaks related with the alpha form in the PCL crystalline phase at 2θ values of 21.5°, 22.0° and 23.9° [40]. PCL-MCC exhibited the characteristic peak of PCL as well as an increase in the intensity of the shoulder at around at 2θ = 22.5° due to the introduction of cellulose, which increased its intensity with an increasing amount of MCC [39]. PCL-PHB electrospun mats displayed two small peaks corresponding to the typical reflection peaks of PHB at 2θ = 13.6° and at 2θ = 16.9° [31,33], which are particularly evident in the PCL90-PHB10 mat due to the higher amount of PHB.

**Figure 7:**
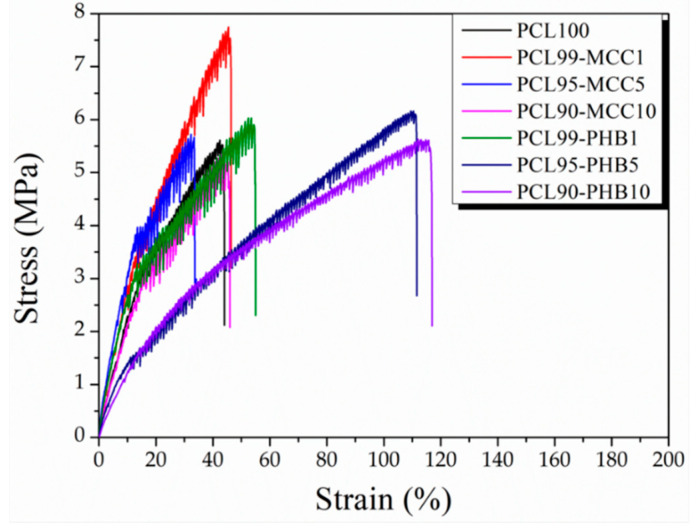
Stress–strain curves for electrospun neat PCL, PCL-MCC and PCL-PHB blends.

Tensile tests were performed in order to obtain mechanical parameters that could give information about the variation of the mechanical properties of the polymeric blends obtained by electrospinning processes. In Figure 7 the stress–strain curves for all the different blends are reported.

Firstly, it is easy to note the loss of ductility of PCL when it is obtained in the form of electrospun fibers. In fact, it is well-known that PCL in its bulk form presents an elongation at break of about 1000% [41], however, when it is obtained in the form of electrospun fibers its behavior is completely different, reaching an elongation at break of about 50%. In this regard we have to point out that woven no-woven electrospun fiber mats are formed by fiber entanglements and voids; it is not a compact material, which strongly affects its mechanical response [16].

**Figure 8:**
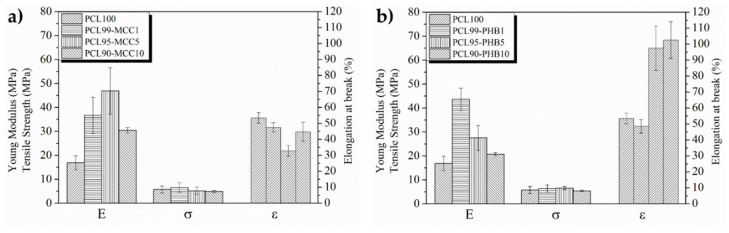
Variation of elastic modulus, tensile strength, and elongation at break for (**a**) PCL-MCC and (**b**) PCL-PHB blends.

Not only is the elongation at break strongly affected by the entanglement fiber structure in the electrospun mat, but also its Young’s modulus (E) and its tensile strength (σ), show values of about 15 and 1 MPa, respectively. However, the behavior of the different blends is completely different, as indicated in Figure 8, where the Young’s modulus, the tensile strength and the elongation at break (ε) for the different electrospun blends are reported. Table 4 summarizes the mean values of Young’s modulus, tensile strength and elongation at break of the different electrospun formulations.

In particular, the PCL-based blends response is different when MCC or PHB are added, mainly in terms of stretchability of the final woven no-woven electospun blends.

All the electrospun blends show increasing mechanical response with respect to neat PCL. While the tensile strength remains quite constant with very small increases with respect to neat PCL (*p* > 0.05), the variation for Young’s modulus and the tensile strength is quite big (*p* < 0.05). For PCL-MCC blends, the addition of 1% and 5% of MCC significantly (*p* < 0.05) increased Young’s modulus from 17.2 MPa for neat PCL to 36.5 and 47.1 MPa, respectively, evidencing a variation of more than 100% with respect to neat PCL. However, when 10 wt % of MCC was added, the reinforcing effect decreased with respect to the addition of 5 wt % of MCC (*p* > 0.05), but staying twice with respect to neat PLC (*p* < 0.05). This result is in good accordance with the high amount of beads observed in the PCL90-MCC10 formulation; such defects consequently led to a detriment of the mechanical performance the electropsun blend. The tensile strength remains quite constant when MCC is added (*p* > 0.05). However, increasing the amount of MCC, the tensile strength slightly decreases without significant differences (*p* > 0.05). The same trend for the elongation at break was observed, indicating that woven no-woven electrospun PCL-MCC blends slightly lost elasticity with respect the neat electrsopun PCL. The result is completely different when PHB is used. In fact, increasing the amount of PHB in the electrospun blend, the elongation at break increases. In particular, by adding 10 wt % of PHB the elongation at break increases twice with respect to neat electrospun PCL (*p* < 0.05). This result is quite interesting. The blends based on PHB show an enhanched mechanical response in terms of the elastic modulus, tensile strength and also elongation at break. According to the literature, the effect of PHB in enhancement on Young’s modulus in PCL-PHB blends has been previously reported in films [42] and the same behavior is observed in our electrospun systems.

## 4. Conclusions

This study was carried out as an initial step towards the use of electrospun PCL-based formulations blended with MCC or PHB for scalable mat production. The electrospun processing conditions were successfully optimized and both PCL-MCC and PCL-PHB were processed under the same conditions as neat PCL. Increasing the amount of the straighter PHB electrospun fibers was obtained with a decrease of beads, which finally disappeared with the higher amount of PHB of 10 wt %. The lower formation of beads in PCL-PHB mats with respect to PCL-MCC mats has been ascribed to the smaller differences between the solubility parameters of PCL with PHB compared with those of the microcrystalline cellulose.

The PCL-MCC and PCL-PHB showed enough thermal stability for the processing conditions as well as for the intended uses such as medical devices, membranes for wound dressing, filtration membranes, films for agricultural or packaging applications, etc. The high amount of hydroxyl groups of MCC were able to positively interact with the PCL matrix, slightly increasing the melting temperature of PCL and also varying the maximum degradation temperature. Although, DCS analysis revealed that PCL and PHB present two melting peaks, the introduction of PHB led to the formation of homogeneous materials and PHB reduced the fiber defects by increasing the amount of PHB. The addition of both MCC and PHB allowed obtaining a stretchable mat with enough thermal stability, high flexibility and improved mechanical response, showing their potential for its use in several industrial applications.

Therefore, it can be concluded that MCC and PHB can be used to tailor the electrospun PCL performance according to the required industrial applications without the need to vary the PCL electrospun processing conditions. Thus, the materials developed here show their potential for scalable mat production for the development of sustainable electrospun industrial products for several fields such as the biomedical, packaging and agricultural sectors.

## Figures and Tables

**Table 1:** Electrospun PCL-based formulations.

Formulations	PCL (%)	MCC (%)	PHB (%)
PCL100	100	-	-
PCL99-MCC1	99	1	-
PCL95-MCC5	95	5	-
PCL90-MCC10	90	10	-
PCL99-PHB1	99	-	1
PCL95-PHB5	95	-	5
PCL90-PHB10	90	-	10

**Table 2:** Optimization of the electrospinning processing parameters and average fiber diameters for neat PCL.

Run	Q_p_ (mL/h)	Q_s_ (mL/h)	V^+^ (kV)	V^−^ (kV)	Fiber Diameter (nm)
I	1	1	1	1	No fiber formation
II	1	1	4	4	150 ± 10
III	1	1	4	7	No fiber formation
IV	1	1	4	10	No fiber formation
V	1	1	7	4	No fiber formation
VI	1	1	7	7	No fiber formation
VII	1	1	7	10	No fiber formation
VIII	1	1	10	4	190 ± 20
IX	1	1	10	7	Beads formation
X	0.5	0.5	10	10	Beads formation
XI	0.5	1	10	10	Beads formation
XII	0.5	3	10	10	No fiber formation
XIII	0.5	5	10	10	210 ± 20
**X**IV	1	0.5	10	10	200 ± 10
**XV**	**1**	**1**	**10**	**10**	**140 ± 10**
**X**VI	1	3	10	10	Beads formation
**X**VII	1	5	10	10	Beads formation
**X**VIII	3	1	10	10	220 ± 10
XIX	3	3	10	10	300 ± 20
XX	5	0.5	10	10	Beads formation
XXI	5	1	10	10	Beads formation
XXII	5	5	10	10	Beads formation

**Table 3:** TGA and DSC thermal parameters of electrospun blends.

Formulations	*T*_5%_ (°C)	*T*_maxPHB_ (°C)	*T*_maxPCL_ (°C)	*T*_gPCL_ (°C)	*T*_mPCL_ (°C)	Δ*H*_mPCL_ (°C)	*T*_mPHB_ (°C)	Δ*H*_mPHB_ (°C)	χ_c_ (%)
PCL100	377.2 ± 0.8	-	419.3 ± 0.6	−62 ± 2	61.4 ± 0.4	79.3 ± 0.5	-	-	53.6
PCL99-MCC1	356.0 ± 1	-	405.4 ± 0.5	−64 ± 3	63.3 ± 0.5	71.1 ± 0.3	-	-	48.5
PCL95-MCC5	319.4 ± 0.6	-	354.1 ± 0.9	−63 ± 1	63.3 ± 0.4	72.0 ± 0.2	-	-	51.2
PCL90-MCC10	355.2 ± 1.1	-	416.2 ± 1.0	−63 ± 1	63.8 ± 0.3	73.0 ± 0.1	-	-	54.7
PCL99-PHB1	376.0 ± 0.7	273.5 ± 0.5	418.3 ± 0.7	−63 ± 1	63.0 ± 0.1	68.2 ± 0.4	172.4 ± 0.6	1.0 ± 0.1	47.2
PCL95-PHB5	287.1 ± 0.3	271.6 ± 0.9	416.1 ± 0.2	−62 ± 2	64.3 ± 0.5	73.1 ± 0.2	173.4 ± 0.5	3.8 ± 0.2	54.7
PCL90-PHB10	255.3 ± 0.9	260.50.7	408.1 ± 0.9	−62 ± 1	64.2 ± 0.3	62.0 ± 0.1	175.7 ± 0.7	6.0 ± 0.5	51.0

**Table 4:** Mechanical response of the different electrospun blends studied here.

Formulations	Young Modulus (MPa)	Variation with Respect to PCL (%)	σ (MPa)	Variation with Respect to PCL (%)	ε (%)	Variation with Respect to PCL (%)
PCL100	17.2 ± 7.2 ^a^	-	5.8 ± 2.3 ^a^	-	52.9 ± 6.6 ^a^	-
PCL99-MCC1	36.5 ± 16.5 ^b,c,d^	212	6.8 ± 3.2 ^a^	117	47.8 ± 4.0 ^a,b^	90
PCL95-MCC5	47.1 ± 15.4 ^b,d^	274	5.2 ± 2.2 ^a^	89	32.0 ± 4.3 ^b^	60
PCL90-MCC10	37.8 ± 4.6 ^b,c,e^	220	4.7 ± 0.9 ^a^	81	44.5 ± 8.4 ^a,b^	84
PCL99-PHB1	42.6 ± 14.3 ^d^	248	6.1 ± 2.1 ^a^	105	48.8 ± 8.5 ^a,b^	92
PCL95-PHB5	32.7 ± 16.1 ^a,c^	190	6.8 ± 1.3 ^a^	117	95.6 ± 19.9^c^	181
PCL90-PHB10	21.2 ± 3.8 ^a,e^	123	5.2 ± 0.9 ^a^	89	98.7 ± 19.3^c^	187

^a–e^ Different superscripts within the same column indicate significant differences between formulations (*p* < 0.05).

## Supplementary Materials

The following are available online at https://www.mdpi.com/1996-1944/13/17/3853/s1. Figure S1. Optical microscopy images at 100× of electrospun PCL fibers of the different run of Table 2 where the beads formation is reported. Figure S2. Fiber diameter distribution of the formulation of Table 2. Figure S3. The corresponding fiber size distribution of the blend formulations. Figure S4. Neat PHB fibers obtaining by electrospinning and their fiber size distribution.
