# Supplementary material for: Electrospinning of PCL-Based Blends: Processing Optimization for Their Scalable Production
Source: Materials (Basel). 2020 Sep 1;13(17):3853. doi: 10.3390/ma13173853 (PMC7504022; doi:10.3390/ma13173853)
Supplement: Supplementary file 1 [file materials-13-03853-s001.pdf]

materials

Supplementary

Electrospinning of PCL-Based Blends: Processing Optimization for their Scalable Production

Marina P. Arrieta [1,2], Adrián Leonés Gil [1,3], Maysa Yusef [1], José M. Kenny [1,4] and Laura Peponi [1,3,*]

1. Instituto de Ciencia y Tecnología de Polímeros (ICTP-CSIC), C/Juan de la Cierva 3, 28006 Madrid, Spain; marrie06@ucm.es (M.P.A.); aleones@ictp.csic.es (A.L.G.); maysay.b@hotmail.es (M.Y.); jose.kenny@unipg.it (J.M.K.)
2. Facultad de Óptica y Optometría, Universidad Complutense de Madrid (UCM), Arcos de Jalón 118, 28037 Madrid, Spain
3. Interdisciplinary Platform for Sustainable Plastics Towards a Circular Economy-Spanish National Research Council (SusPlast-CSIC), 28006 Madrid, Spain
4. Civil and Environmental Engineering Department, STM Group, University of Perugia, 05100 Terni, Italy

* Correspondence: lpeponi@ictp.csic.es

Received: 30 July 2020; Accepted: 28 August 2020; Published: date

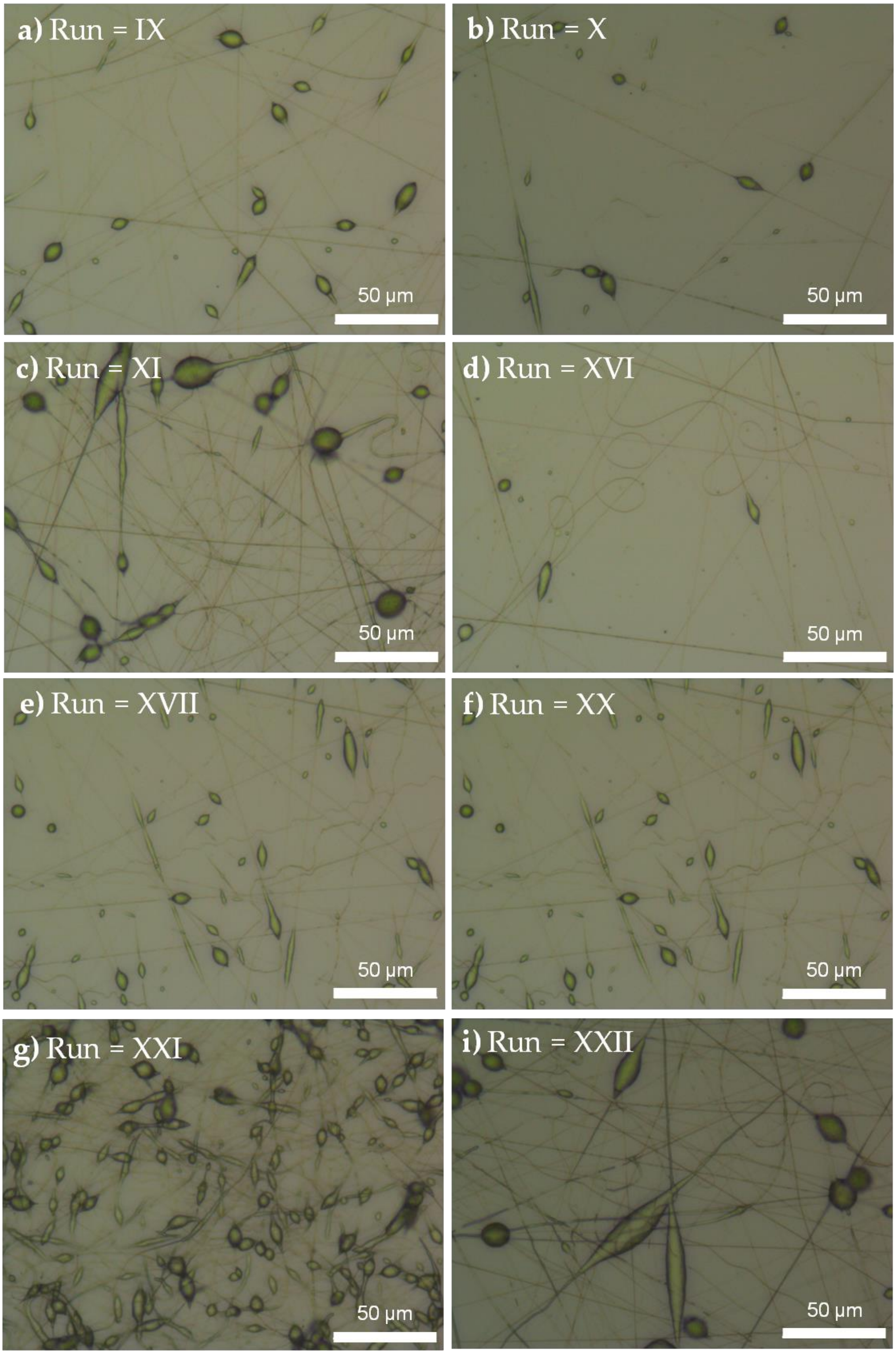

Figure S1. Optical microscopy images at 100 × of electrospun PCL fibers of the different run of Table 2 where the beads formation is reported. (**a**) Run IX, (**b**) Run X, (**c**) Run XI, (**d**) Run XVI, (**e**) Run XVII, (**f**) Run XX, (**g**) Run XXI and (**h**) Run = XXII.

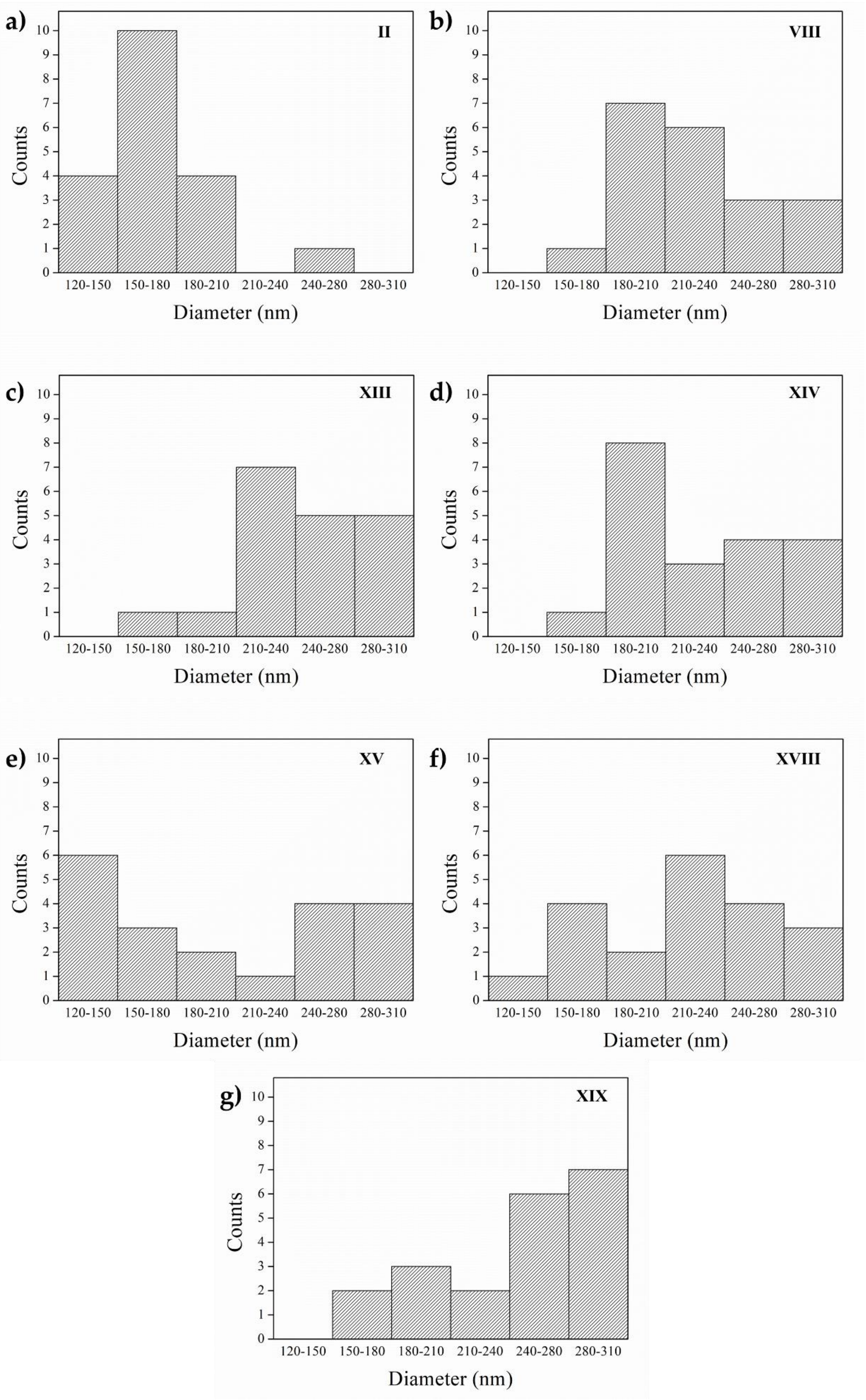

Figure S2. Fiber diameter distribution of the formulation of Table 2.

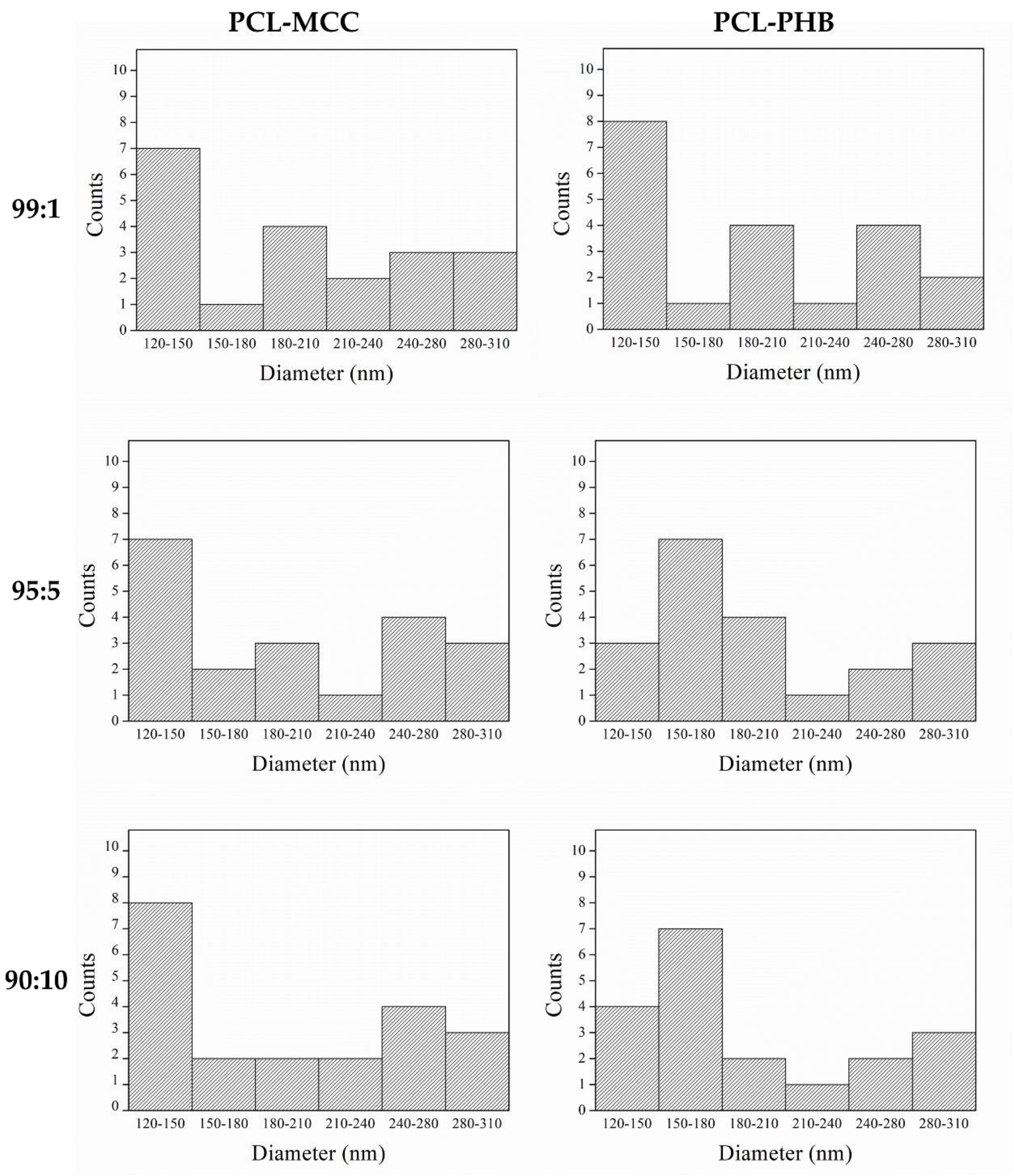

Figure S3. The corresponding fiber size distribution of the blend formulations.

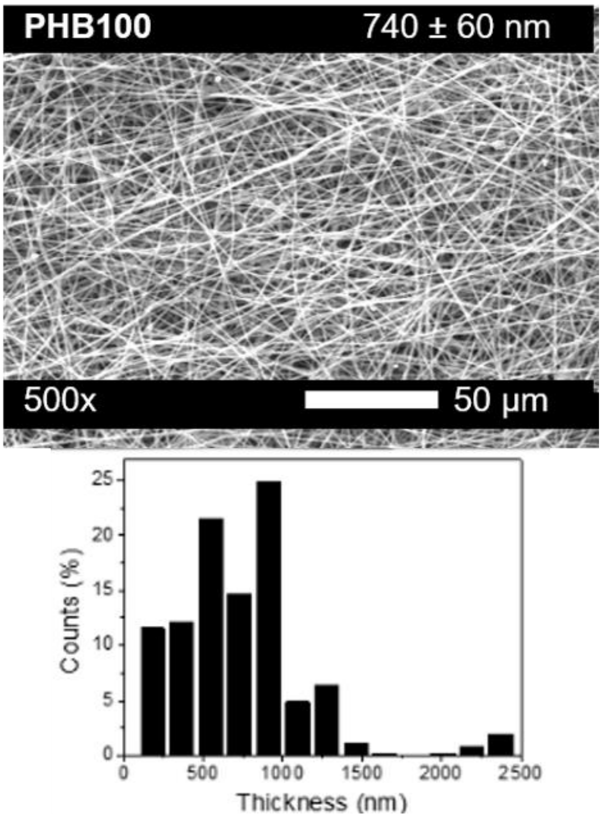

Figure S4. Neat PHB fibers obtaining by electrospinning and their fiber size distribution.

© 2020 by the authors. Submitted for possible open access publication under the terms and conditions of the Creative Commons Attribution (CC BY) license (http://creativecommons.org/licenses/by/4.0/).
